# The Vaginal Microbiome and Recurrent and Chronic Urinary Tract Infection

**DOI:** 10.1007/s00192-025-06434-1

**Published:** 2025-12-08

**Authors:** Katie L. Dalby, Harry Horsley, David Spratt, Rajvinder Khasriya

**Affiliations:** 1 Department of Microbial Diseases, Eastman Dental Institute, University College London, London, NW3 2PF UK; 2 Centre for Kidney and Bladder Health, University College London, London, NW3 2PF UK

**Keywords:** Genitourinary microbiome, Menopause, Pelvic pain, Urinary tract infection, Vaginal microbiome, Vaginal pain

## Abstract

**Introduction and Hypothesis:**

The vaginal and urinary microbiomes are closely linked, yet the role of this relationship in recurrent and chronic urinary tract infections (UTIs) remains uncertain. Research into genitourinary ecology and UTI has largely focused on acute infections and reproductive age groups, leaving a gap in understanding the role of the microbiome in recurrent and chronic cases. This review is aimed at presenting that disruptions within vaginal microbiota contribute to UTI chronicity in menopausal women, highlighting the potential for microbiome-targeted interventions in this high-risk group.

**Methods:**

A comprehensive literature review was conducted, with search terms including cystitis, urogenital, antibiotic, infection, and bladder, vaginal microbiome, vaginal ecology, topical oestrogen, atrophy, genitourinary syndrome of menopause, vulvo-vaginal atrophy (VVA), bacterial vaginosis and lactobacillus. Relevant articles were screened, critiqued, and synthesised based on key themes.

**Results:**

*Lactobacillus* spp. appears to be the key component of a healthy vaginal microbiome. Decreased vaginal *Lactobacillus* abundance, seen with vaginal dysbioses and the menopause, correlates with an increased presence of urinary pathogens, increasing susceptibility to UTI. This review demonstrates that interventions to optimise vaginal ecology could reduce UTI burden. These approaches offer non-antibiotic treatment strategies, lowering antimicrobial resistance risk. However, studies frequently exclude those with chronic and recurrent infections, underscoring the necessity for more research targeting this group.

**Conclusions:**

This review highlights the link between disrupted vaginal ecology and recurrent and chronic UTI, and the need for expanded research into microbiome-targeted treatments. A paucity of studies researching recurrent and chronic UTI cohorts limits the evidence base for clinical generalisation, meaning that more focussed studies are needed to improve understanding and clinical management.

## Introduction

The urinary tract microbiome is now recognised as diverse and dynamic, challenging the historical paradigm of urinary tract sterility [1]. This paradigm shift has catalysed research into urinary tract infection (UTI) pathophysiology, addressing a condition that represents a substantial clinical and economic burden to health care systems [2]. Women are far more likely to suffer from UTI than men, with a lifetime incidence of over 50% [3]. Furthermore, the menopausal state increases the risk of recurrence [4] and this group make up a significant proportion of patients who develop recurrent and chronic infections. The European Association of Urology Guidelines (EUA), cited by those from the National Institute for Health and Care Excellence (NICE), defines recurrent UTI as two or more UTI episodes in 6 months or three or more UTIs in 12 months [5]. In specialist UTI services, patients frequently report persistent symptoms accompanied by ongoing pyuria, warranting the designation “chronic UTI” [6]; a formal diagnosis is difficult to achieve and often takes years.

The pathophysiology of chronic UTI is complex. The presence of diverse bacteria in the urine of even asymptomatic individuals poses a challenge in identifying “pathogens”, further complicated by the presence of overlapping bacterial species in both symptomatic patients and those with no UTI history. More definitively established is the protective role of *Lactobacillus* spp., which are one of the most abundant commensal bacteria identified in the bladder of healthy individuals [7], with experimental evidence demonstrating that *Lactobacillus* exposure reduces *Escherichia coli* levels—the most common uropathogen—in murine bladders [8]. *Lactobacillus* is well known for its role in the vaginal microbiome, as the predominant bacterial genus of a typical healthy vaginal environment [9], but a multitude of factors such as medication or ageing can disrupt this. Such disruption results in conditions such as bacterial vaginosis (BV); characterised by a raised vaginal pH, loss of *Lactobacillus* and an overgrowth of multiple bacteria such as *Gardnerella* and *Mobiluncus*, potentially but not always manifesting with offensive vaginal discharge, vaginal discomfort or itch and burning on urination [10]. Given these established relationships, it seems a logical research priority to explore the potential role of vaginal ecology in the UTI health-disease axis. Indeed, research employing sequencing techniques on paired urine and vaginal samples has shown an overlap of the urinary and vaginal microbiomes, with the conclusion that the two act as biological niches for bacteria and directly influence one another [11–13]. A study performing real-time quantitative polymerase chain reaction (qPCR) in paired urine and vaginal samples further strengthened this argument, showing that vaginal abundance of specific *Lactobacillus* species was directly related to its abundance in the urine [13], whereas other research has shown that the vaginal microbiota of women suffering with recurrent UTI is more likely to contain uropathogens such as *E. coli* than women without a UTI history [14].

The growing recognition of the vaginal microbiome in urogenital disease, particularly UTI, represents an important research frontier. If *Lactobacillus* can prevent infection with common urinary pathogens in the bladder, then the manipulation of the vaginal niche as a key source of urogenital lactobacilli may optimise not only vaginal but also bladder health, and is an avenue worth researching. This article is aimed at summarising the current evidence base for the role of the vaginal microbiome in recurrent/chronic UTI, focussing on high-risk groups such as menopausal women, and describes potential areas for further research.

## Review of Current Evidence

We searched MEDLINE, EMBASE, Web of Science, and Clinicaltrials.gov from inception until 2024 as part of a focused literature review, along with a manual search of the reference lists of relative studies to retrieve articles that responded to the identified objectives (Table 1). Search terms included, but were not limited to cystitis, urogenital, antibiotic, infection, and bladder, vaginal microbiome, vaginal ecology, topical oestrogen, atrophy, genitourinary syndrome of menopause (GSM), vulvo-vaginal atrophy (VVA), bacterial vaginosis, lactobacillus.

**Table 1:** Vaginal microbiome + post-menopausal urinary tract infection review

Reference	Year	Study population	Menopausal status	Study design	Analysis techniques	Major themes	Strengths	Weaknesses
Berard et al. [15]	2023	405 African women aged 18–51	Pre-menopausal	Experimental (in vivo and in vitro)	Multi-omics	Characterisation of genital tract processes underlying BV	Large sample size, advanced methodology	Complex and very specific, hard to generalise results or apply in vivo
Boskey et al. [16]	2001	11 reproductive-age women	Pre-menopausal	Experimental (in vivo)	Lactate analysis	Origins of vaginal lactic acid	Detailed focus	Small sample size
Eschenbach et al. [17]	1989	67 women with BV	Pre-menopausal	Cross-sectional	Vaginal swab culture	Hydrogen peroxide-producing *Lactobacillus*	Key scientific evidence for *Lactobacillus* promoting vaginal health	Methods may now be outdated
Stapleton et al. [18]	2011	100 pre-menopausal women with recurrent UTI	Pre-menopausal	RCT	16S rRNA gene qPCR of vaginal samples	Effect of *L. crispatus* probiotic on UTI prevention	Clinical and microbial outcomes	No comparison with conventional treatment; menopausal population excluded
Czaja et al. [19]	2007	30 pre-menopausal women	Pre-menopausal	Phase I trial	Safety and tolerability analysis	Safety of *L. crispatus* vaginal probiotic pessary	First-in-human safety data evidence	Small sample size, no efficacy outcomes
Taylor-Robinson et al. [20]	2002	100 post-menopausal women	Post-menopausal	Cross-sectional	Gram stain and grading of vaginal flora	Vaginal flora composition in post-menopausal women	Focus on vaginal health post-menopause	Descriptive results only
Geng et al. [21]	2020	100 post-menopausal Chinese women	Post-menopausal	Cohort study	Vaginal 16S rRNA sequencing	Impact of HRT on vaginal microbiota	Longitudinal follow-up, good sample size, thorough methodology	Focus on GSM with UTI as subset of symptoms
Mitchell et al. [22]	2018	Post-menopausal women with GSM	Post-menopausal	RCT (subset analysis)	Vaginal 16S rRNA sequencing; symptom monitoring	Vaginal microbiota and GSM symptoms during treatment vs placebo	RCT with combined microbial and clinical data	Subset analysis only, no specific clinical UTI outcomes
Srinivasan et al. [23]	2022	144 post-menopausal women	Post-menopausal	RCT (secondary analysis)	Metabolomics and microbiome profiling	Impact of topical oestrogen on vaginal microbiota	Sophisticated methods, both metabolomic and microbiome data	Secondary analysis, no UTI symptom outcome measures
Kirkengen et al. [24]	1992	40 post-menopausal women	Post-menopausal	RCT	Symptom monitoring	Effect of oral oestriol on UTI prevention	RCT in target population	Small sample, methods not clearly detailed and possibly outdated
Eriksen [25]	1999	108 post-menopausal women	Post-menopausal	RCT	Symptom monitoring; vaginal pH	Effect of vaginal estradiol ring on UTI, GSM, and pH	Well-designed, includes subjective outcomes and objective measures of vaginal health	No microbial data, methodology possibly outdated (UTI confirmed by culture alone)
Raz and Stamm [26]	1993	93 post-menopausal women with recurrent UTI	Post-menopausal	RCT	UTI incidence; vaginal and urine cultures; vaginal pH	Effect of intravaginal estriol on UTI prevention	Well-designed, long duration; clinical and microbial outcomes	Outdated methodology (vaginal flora and UTI presence assessed by culture)
Beerepoot et al. [27]	2012	252 post-menopausal women with recurrent UTI	Post-menopausal	Double-blind RCT	Symptom monitoring; UTI incidence; antimicrobial resistance	*Lactobacillus* vs antibiotics for UTI prevention	Large RCT comparing novel with conventional treatment	Narrow assessment of vaginal flora limits clinical relevance
Sekito et al. [28]	2023	39 post-menopausal women	Post-menopausal	Observational + interventional subset	Vaginal 16S rRNA sequencing	Etiology of recurrent UTI and role of probiotics	Combined observational + interventional data	Small sample size, retrospective
Lillemon et al. [29]	2022	39 post-menopausal women	Post-menopausal	RCT	16S rRNA sequencing of urine and vaginal samples	Effect of local estrogen on GSM and microbiome	RCT, advanced methodology	Small sample size, asymptomatic population
Gupta et al. [30]	1998	65 women with recurrent UTI and 75 controls	Mixed	Observational (case–control)	Vaginal swab culture	Relationship between vaginal *E. coli* and *Lactobacillus*	Case–control design, novel hypothesis	Culture-based methods now outdated, mixed menopausal status
Ray Mohapatra, Jeevaratnam [31]	2019	Laboratory in vitro models	Mixed	Experimental (in vitro)	Antibiofilm and bacterial activity assays	Antibacterial activity of *Lactobacillus* bacteriocins	Targeting a key clinical issue, novel concept	In vitro, limits human relevance
Brannon et al. [32]	2020	In vitro models; 4 women with recurrent UTI	Mixed	Experimental (in vitro and ex vivo)	Cell invasion assays	*E. coli* invasion of vaginal epithelium	Strong research concept focussing on host factors	Small in vivo cohort with little demographic information
Yoshikata et al. [33]	2022	70 women (35 pre-menopausal, 35 post-menopausal)	Mixed	Pilot RCT	Vaginal 16S rRNA sequencing; vaginal pH; clinical symptoms	Effect of *Lactobacillus*-containing hygiene products on GSM	Objective and subjective patient data points; modern sequencing methods	Pilot trial, power calculation doubtful; no inclusion of UTI as an outcome within GSM
Zeng et al. [34]	2024	96 women aged 18–97 years	Mixed	Observational with interventional subset	Vaginal 16S rRNA sequencing; symptom monitoring	Relationship between microbiota and GSM; probiotic impact	Observational and interventional data, inclusion of peri-menopausal subset	Heterogeneous sample, no post-menopausal controls

*BV* bacterial vaginosis, *RCT* randomised controlled trial, *rRNA* ribosomal RNA, *qPCR* quantitative polymerase chain reaction, *UTI* urinary tract infection, *HRT* hormone replacement therapy, *GSM* genitourinary syndrome of menopause

## Defining a Healthy Vaginal Microbiome

Research into the vaginal microbiome began over 100 years ago [35] with the discovery that *Lactobacillus* dominates a healthy vaginal microbiome established shortly thereafter. The poly-microbial dysbiosis of BV was also described, although not with our current understanding [35]. Further work revealed that a healthy vaginal microbiome undergoes significant changes throughout the female lifespan. The low-oestrogen state of infancy is characterised by a diverse microbiome comprising anaerobes, *Staphylococci*, diphtheroids, *E. coli* and *Mycoplasma* spp. This remains the case during early childhood until menarche, when circulating oestrogen and progesterone transform the microbiota into the *Lactobacillus*-dominated state defining the reproductive years [36].

Research focussing on lifespan assessment of the microbiome has demonstrated that *Lactobacillus* levels decline with advancing age, even in healthy women reporting no vaginal symptoms [20]. This increased diversity and loss of *Lactobacillus* predominance is consistent after the menopause, a seminal event in the female lifespan often overlooked in research. It is largely agreed that this is mediated by the loss of circulating oestrogen, a hormone that increases levels of intracellular glycogen at the vaginal epithelium, subsequently metabolised to lactic acid, lowering vaginal pH and creating a beneficial environment for vaginal eubiosis. Lactobacilli contribute additionally by producing lactic acid and hydrogen peroxide, thought to prevent *E. coli* colonisation of the vagina and bladder [30]. The antimicrobial activity of bacteriocins, toxins produced by lactic acid bacteria such as *Lactobacillus*, have even been posited as an alternative to antibiotics due to their broad-spectrum effect at both acidic and neutral pH. Work by Ray Mohapatra et al. explored the potential clinical use of bacteriocins in the context of UTI; when applied as a coating on urinary catheters, bacterial adherence and biofilm inhibition were reduced, which could prevent this common and often serious healthcare-associated infection [31]. These findings support the role of *Lactobacillus* in the healthy urogenital tract, protecting not only against vaginal disease but also against common UTI pathogens—something that is lost after the menopause [16, 17]. Although oestrogen supplementation demonstrates efficacy in reducing UTI burden in post-menopausal women, evidence remains limited. A Cochrane review identified only two randomised controlled trials (RCTs) [37] and definitive mechanistic evidence of oestrogen’s protective effect in this context is lacking.

Although the loss of vaginal lactobacilli and increased microbial diversity after the menopause is not inherently pathological, the potential link between these changes and an increase in the prevalence of urogenital disease is being investigated. Vulvovaginal atrophy (VVA) occurs exclusively in the menopausal state, coinciding with UTIs becoming more complicated and more prone to chronicity. GSM is the updated term for VVA and encompasses a broader range of symptoms including vaginal dryness, pain on sex or touch, and painful and frequent urination—a clinical presentation overlapping substantially with recurrent/chronic UTI. A negative relationship between Lactobacillus dominance and GSM and/or VVA has been successfully demonstrated [21, 22]; however, oestrogen supplementation does not consistently improve both *Lactobacillus* dominance and symptoms in tandem [23], indicating that the underlying mechanisms are more complex than initially anticipated.

Subsequent research revealed associations between disrupted vaginal microbiota and pathology including UTI [38] but also miscarriage, preterm birth, cervical neoplasm [39] and sexually transmitted infections including Human Immunodeficiency Virus [10]. However, studies often reveal conflicting results, with no obvious pathway to prevention of pathology. Much of this research has focussed on women of childbearing age, particularly the gold-standard scoring systems we use to diagnose BV today [10, 40], which leads to much uncertainty about the prevalence, diagnosis and treatment of vaginal symptoms and their potential sequelae or associations in a post-menopausal population [41].

Interestingly, associations between BV and UTI have been demonstrated [38, 42, 43], which strongly suggests that vaginal dysbiosis might be a predisposing factor. However, although the majority of women display a typical *Lactobacillus*-dominated microbiome, many asymptomatic women have a diverse microbiota [9] and almost all menopausal women lack *Lactobacillus* in the absence of BV or other pathology [20]. Therefore, more investigation is needed to define a ‘healthy’ vaginal microbiome and how it may protect or weaken host defences in the urogenital tract. Looking to answer this question, multi-omics research has suggested that vaginal dysbiosis might interrupt key cell-signalling pathways and their metabolites, leading to epithelial inflammation and barrier dysfunction [15], which implies that it is not simply the presence or absence of certain microbiota but also microbiome signalling pathways that generate inflammation.

## The Vaginal Microbiome and Urinary Tract Infection

Bacterial reservoirs are accepted as a potential source of recurrent or intractable UTI and represent an active area of research. Adherence assays performed on vaginal epithelial cells obtained from vaginal samples and co-incubated with *E. coli* demonstrated the active bacterial invasion of the genitourinary tract [32]. After transurethral inoculation of *E. coli* in murine models, titres of *E. coli* within vaginal epithelial cells were comparable with those in the bladder. After 4 weeks of observation this presence persisted, with immunofluorescence microscopy of vaginal epithelial cells confirming adherent *E. coli* bacteria, a finding similarly observed in human cells during acute infection. Furthermore, vaginal inoculation of mice with *E. coli* resulted in significant bacteriuria demonstrating that uropathogens of vaginal origin can cause UTI. Finally, fluorescence microscopy of vaginal epithelial cells from women with a history of recurrent UTI confirmed the presence of intracellular *E. coli*. This research collectively establishes the capacity of bacteria to invade vaginal epithelial cells during the acute phase of a UTI and persist as a reservoir.

Early in vivo studies established the safety and efficacy of vaginal oestrogen for UTI prevention. A randomised controlled trial found that intravaginal oestrogen administered over 12 weeks reduced UTI frequency and vaginal pH in the intervention arm with statistical significance, with no adverse events reported. However, this study did not assess changes in the vaginal or urinary microbiota. Similarly, in 1999, Eriksen recruited 108 women with a history of recurrent UTI and assigned them to either an intravaginal oestrogen ring or no oestrogen treatment. Over 36 weeks of follow-up, vaginal pH and symptoms were monitored [25], demonstrating a statistically significant reduction in vaginal pH and time to UTI relapse in the intervention group. Although these studies confirmed clinical benefits, neither assessed vaginal or urinary microbiota, leaving the mechanistic link between pH reduction and increased lactobacilli unproven.

Research into the role of the vaginal microbiome in recurrent and chronic UTI has since expanded. In 1993, Raz and Stamm [26] conducted a pivotal 8-month RCT studying whether intravaginal oestrogen cream compared with placebo could prevent UTI. The intervention group showed a significant reduction in UTI flares, as well as a change in the microbiome; *Lactobacillus* increased in presence within samples from zero at baseline to 58% (21 out of 36) after treatment, whereas Enterobacteriaceae colonisation more than halved. The authors attributed UTI reduction to a modification in vaginal flora. Despite the limitations of the urine culture, now known to be flawed, this remains one of the few RCTs examining vaginal ecology in post-menopausal women with recurrent UTI.

An observational study of women undergoing pelvic floor repair further validated these findings [10]. In this largely menopausal population, bladder microbiomes deficient in *Lactobacilli*—particularly *L. iners*—showed greater microbial diversity, more uropathogens, and higher postoperative UTI rates.

Subsequent research supported the protective role of *Lactobacillus*. A case–control study found that vaginal *E. coli* colonisation was significantly more common in women with a UTI history than in controls [30]. Results showed that crucially, women lacking hydrogen peroxide-producing *Lactobacillus* were significantly more likely to harbour vaginal *E. coli*, supporting the theory that *Lactobacillus* suppresses this pathogen [11]. These results highlight the need for further research into the effect of vaginal oestrogen on the genitourinary microbiome and clinical UTI burden in a post-menopausal population. These studies demonstrate the evolution from basic clinical outcomes to a pathophysiological understanding of the protective effects of oestrogen.

Stapleton et al. directly tested lactobacilli supplementation in pre-menopausal women with active, uncomplicated UTIs and history of recurrence [18, 19]. Researchers randomised participants to *L. crispatus* pessaries or placebo for ten weeks, treating any UTI episodes with standard antibiotics without interrupting study treatment. Among 87 women, 93% in the treatment group achieved high-level *L. crispatus* colonisation (defined by 16S rRNA gene sequencing) versus 68% with placebo—a significant difference. Crucially, probiotic use combined with high-level colonisation reduced UTI frequency by approximately 50%. Interestingly, when placebo participants naturally achieved high-level *L. crispatus* colonisation, they experienced no UTI benefit. These findings question whether lactobacilli-rich vaginal microbiomes naturally prevent UTIs in pre-menopausal women but suggests that active optimisation of the vaginal microbiome might benefit those with active and recurrent UTIs, particularly alongside antimicrobial treatment. This study excluded post-menopausal women and ‘complicated’ UTI cases, limiting applicability to a recurrent/chronic UTI population. Beerepoot et al. (2012) compared oral *L. reuteri* probiotics with trimethoprim-sulfamethoxazole prophylaxis in 252 post-menopausal women over 12 months [27]. The study failed to demonstrate the non-inferiority of probiotics for UTI prevention. Researchers detected no *L. reuteri (qPCR)* in vaginal samples after treatment, and Nugent scores showed no improvement. The study's focus on detecting only the probiotic organism limited broader microbiome assessment, and multiple dropouts owing to gastrointestinal disturbance suggested that oral administration might be suboptimal—*L. reuteri* was found in gut samples, indicating that vaginal delivery may improve both efficacy and tolerability.

A 2022 RCT tested *Lactobacillus*-containing vaginal washes and gels in 70 healthy women (35 pre-menopausal and 35 post-menopausal) [33]. Post-menopausal participants showed significantly lower baseline *Lactobacillus* abundance, higher microbial diversity and more genitourinary symptoms than pre-menopausal women (60% vs 34%). After 4 weeks, post-menopausal women in the treatment arm experienced significant improvement in genitourinary symptoms, including overactive bladder scores. The group using *Lactobacillus* gels and washes together showed the greatest benefit, with significant reductions in pathogenic microbiota and vaginal pH. However, neither treatment group achieved statistically significant improvements in pH, *Lactobacillus* abundance or diversity indices compared with controls on 16 s rRNA sequencing, suggesting that washes and gels might deliver insufficient *Lactobacillus* doses. The study's exclusion of women with a UTI history limits applicability to target populations.

A 2023 study specifically examined post-menopausal women with recurrent UTIs using *L. crispatus* probiotics [28]. Researchers compared vaginal microbiomes across healthy controls, acute UTI and recurrent UTI groups. Post-menopausal women with recurrent UTIs showed significantly different vaginal microbiomes—specifically *Lactobacillus* deficiency and Enterobacteriaceae overgrowth—suggesting that vaginal dysbiosis might play a role in recurrent UTI. *L. crispatus* supplementation reduced UTI incidence in the prevention group, although treated microbiomes shifted toward but did not fully match healthy controls, indicating that *Lactobacillus* presence alone is not completely protective.

The studies discussed above demonstrate the varied evidence for the use of probiotics, with a probable advantage of local over oral administration. It can be agreed that vaginal *Lactobacillus* pessaries appear safe and well tolerated, and that it may be beneficial in preventing or reducing UTI in those women with lactobacillus-deficient microbiomes. Sequencing data suggest that vaginal probiotics favourably alter vaginal ecology but do not always completely correct dysbiosis. The lack of protection from naturally occurring high lactobacilli levels indicates multifactorial optimisation requirements [18]. Future research should compare probiotics with indirect *Lactobacillus*-enhancing treatments targeting pH or other vaginal environmental factors.

## The Vaginal Microbiome and Genitourinary Syndrome of Menopause

While UTI-focused vaginal microbiome research remains limited, substantial evidence exists for genitourinary syndrome of menopause (GSM). GSM represents the broad range of symptoms during and after menopause, including those of the lower urinary tract. The nature of this condition suggests oestrogen deficiency at its heart but since not all oestrogen-deficient menopausal women develop GSM, researchers are increasingly examining the vaginal microbiome due to its evidenced change across the female lifetime [44]. In this review, a small number of such studies detailing urinary symptoms in their data collection and analysis have been included.

Lillemon et al. randomised 39 post-menopausal women to a vaginal oestrogen ring (Estring) or a placebo ring over the course of 12 weeks, evaluating vaginal pH, Vaginal Maturation Index (VMI), symptom and clinical scores and vaginal and urinary microbiomes before and after treatment [29]. Any woman not already using HRT was eligible and the cohort was found to be broadly asymptomatic at baseline. The largely asymptomatic cohort showed no significant microbiome differences in an intention-to-treat analysis, but secondary analysis revealed increased VMI and *Lactobacillus* abundance in the treatment group. Urinary microbiomes remained the same. There was also no significant change in symptom scores, but this was most likely due to the population being minimally symptomatic at baseline, and VMI scores improved in the Estring cohort as expected with effective oestrogen therapy. The robust methodology would benefit from larger, symptomatic populations.

A 2020, cross sectional study compared 50 women on tibolone HRT for ≥ 12 months with 50 untreated controls [21]. Symptom surveys, gynaecological examination for vaginal health index scores (VHIS) and vaginal sampling for 16 s rRNA sequencing were undertaken. Although urinary symptoms were included in the GSM symptom assessment, only a minority reported symptoms such as dysuria or recurrent UTI at baseline, although 78% did report vulvovaginal ‘burning’, a symptom often reported by recurrent/chronic UTI patients. HRT users showed improved GSM symptoms, reduced alpha diversity, and greater *Lactobacillus* dominance. *Lactobacillus* correlated negatively with GSM severity and positively with vaginal health scores. Atrophy was associated with pathological bacteria including *Chlamydia*, *Escherichia–Shigella*, and *Streptococcus*. This study further corroborates the efficacy of HRT for GSM and its association with increasing *Lactobacillus* abundance and reduced alpha diversity within the vaginal microbiome. It did not demonstrate any significant benefit for lower urinary tract symptoms, possibly due to mild baseline symptoms or systemic rather than local treatment [45–47].

A recently published cross-sectional study of 96 women aged 18–76 found 83% of peri-/post-menopausal participants had GSM signs and symptoms [34]. Clinical symptom scores for GSM, incontinence, sexual function and quality of life were taken and examinations performed to assess for VVA using the VHIS. Vaginal swabs were taken and 16S rRNA gene sequencing performed to identify microbiota at the species level. Results confirmed that *Lactobacillus* abundance reduces and alpha diversity increases with the menopause, and these changes are significantly associated with the presence of GSM. Furthermore, they established that these statistically significant differences in microbiota correlated significantly with the severity of GSM: participants reporting two or three GSM symptoms showed greater alpha diversity and less *Lactobacillus* abundance than those reporting only one. Specific genera (*Streptococcus*, *Anaerococcus*, *Escherichia–Shigella*, *Enterococcus*) were associated with GSM for genital or sexual but not urinary symptoms; this may have been because the ICIQ-SF questionnaire was used which focusses on leaking of urine rather than dysuria or frequency. A subset of 29 women receiving a 10-day course of *Lactobacillus* probiotic pessaries showed significant genital and sexual symptom improvement but non-significant urinary improvement. Some secondary analyses of participants’ serum oestrogen levels showed no correlation to symptoms, reflecting the favoured theory that increasing local levels of oestrogen is vital in treating GSM and recurrent UTI. These studies consistently demonstrate oestrogen's efficacy for GSM and successful vaginal *Lactobacillus* repopulation, but few adequately assess urinary symptoms or UTI burden at sufficient scale for significance.

## Areas for Further Research

### Current Knowledge Gaps

Limited data exist regarding normal versus pathological states in the vaginal cellular environment and microbiome as they relate to UTI development. Additionally, outcome data on vaginal oestrogen therapy for UTI prevention remain sparse, particularly studies with endpoints focused on urogenital microbiota changes. Cross-sectional sampling in clinical settings is essential to establish a comprehensive baseline understanding of the differences in the vaginal environment and any changes in the ecological patterns in post-menopausal women, as well as subsequent changes following interventions such as antibiotic or oestrogen therapy.

### Oestrogen and Non-Hormonal Vaginal Therapies

Research studying the vaginal microbiome in the context of urinary symptoms is becoming more common but fewer studies are being undertaken in target populations with UTI. A majority of UTI studies focus on *Lactobacillus* probiotics, often alongside antibiotics, but the evidence base remains small and would benefit from further studies to strengthen it. As identified by a previous Cochrane review into oestrogen for recurrent UTI [37], there is a paucity of trials assessing management strategies such as oestrogen. This is particularly relevant because vaginal oestrogen remains an off-label use for recurrent UTI in a post-menopausal population, even though it is now included in national guidance [48] and well-demonstrated to be effective for conditions with symptom overlap such as GSM [21].

The positive role of *Lactobacillus* in the vaginal microbiome is well established and its potential link to bladder health has been discussed thoroughly in this review. A crucial part of this relationship is how closely *Lactobacillus* is linked to vaginal pH. The optimal vaginal pH is generally agreed to be between 3.5 and 4.5 [9] and is abnormally raised in symptomatic vaginal conditions with disrupted vaginal microbiota such as BV, and also in oestrogen deficiency and VVA after the menopause [49–51]. If vaginal dysbiosis could create a vulnerability to infections in the urinary tract, then all treatment strategies for this should be considered. Many women purchase boric acid, a weak acid administered as a vaginal pessary that is an approved treatment for vulvo-vaginal candidiasis and recurrent vaginitis with growing evidence for other infections such as *Trichomonas vaginalis* [52, 53]. It is thought to work by lowering vaginal pH, which also disrupts cell membranes with a bacteriostatic and fungistatic effect with the potential to disrupt pathogenic vaginal biofilms [54] including *Candida* [55]. Its success in treating vaginal infections and its effect on vaginal pH could translate into management of UTI owing to the now proven overlap between bladder and vaginal microbiota, but to our knowledge there are no studies assessing this as yet.

### Recruiting Symptomatic Participants and Study Design

Before looking to UTI, it would be remiss not to acknowledge the historic lack of funding for research specific to women’s health. This so-called “gender gap” in research is now being addressed, but the reproductive ages remain a priority, with menopause lagging behind [56, 57]. Women with pre-existing urinary symptoms or those already on long-term treatment for UTI are almost always excluded from relevant studies, which means that the complex recurrent/chronic UTI population is presently under-studied with regard to the vaginal microbiome. Effective research in this field requires recruitment from specialist clinics within secondary and tertiary care services, ideally in collaboration with primary care providers. National multicentre studies are essential, and research should be concentrated in Urology and Urogynaecology centres with specific UTI expertise. These populations are difficult to reach outside specialist settings owing to the prolonged time to diagnosis and limited awareness of these conditions. Cross-sectional study designs offer significant ethical advantages by enabling pragmatic data collection from patients at clinic entry (prior to treatment initiation) and from those already established on therapy regimens. This approach facilitates meaningful comparisons without withholding or discontinuing existing treatments, thereby avoiding the ethical concerns inherent in longitudinal studies potentially requiring treatment interruption.

Although randomised controlled trials remain the gold standard, this review highlights a critical lack of empirical research in post-menopausal populations with complex UTI. High-quality studies employing alternative methodologies would therefore be valuable. Given recruitment challenges for treatment-naive patients, pragmatic studies comparing genitourinary microbiota between patient cohorts and matched controls could provide an excellent foundation for future work.

One limitation of any research into UTI is the controversy surrounding diagnosis of UTI episodes; as previously mentioned, standard urine culture is thought by many UTI specialists to be insufficiently sensitive and an inferior method to fresh urine microscopy. Study endpoints should prioritise clinically meaningful outcomes including symptom control (particularly pain management), prevention of sepsis and hospital admissions, and patient-reported improvement measures consistent with other research into chronic conditions.

## Conclusion

Current evidence suggests that the urinary and vaginal microbiomes might be inextricably linked. The vaginal microbiome is proven to change across the female lifespan in line with the hormonal state, and can be modulated by hormones, probiotics and other medications. The management of the urogenital microbiome in UTI remains an emerging field, particularly in older women with recurrent or chronic symptoms. However, the studies summarised in this review show evidence of an altered post-menopausal vaginal microbiome in the context of complex UTI and a clinical reduction in UTI flares and symptoms with treatments such as vaginal oestrogen, with associated decreases in vaginal pH and increased detection of *Lactobacillus*. This increase in the abundance *Lactobacillus* species within the vaginal microbiome appears to stimulate increased *Lactobacillus* abundance within the bladder in turn, and be protective against UTI. There has been good progress in studying the efficacy and safety of *Lactobacillus* repopulation within the urogenital microbiome with promising results. Probiotic administration has been shown to increase *Lactobacillus* abundance and reduce UTI burden and although this effect has been variable across different trials, it is a safe and well-tolerated intervention that generates no antimicrobial resistance. Further work is needed to confirm the processes by which these agents are protective against UTI to enable more widespread use.

Treatment of UTI continues to be dominated by antibiotics, meaning that the importance of the vaginal microbiome is twofold: cause, but also effect. Specialist management of recurrent and chronic infection often includes long-term antibiotic use, which has potential drawbacks such as unpleasant side effects or vulvo-vaginal candidiasis, but also more serious consequences such as *Clostridium difficile* infections or the potential for antimicrobial resistance. The full impact of this may be yet to manifest, as the cohort of patients on long-term therapeutic regimes is small. This poses a strong argument for a larger evidence base for non-antibiotic UTI treatment strategies, including oestrogen and *Lactobacillus* probiotics, but also the potential use of treatments for vaginal dysbiosis such as boric or lactic acid [58]. Additionally, although such antibiotic regimes are often instrumental in relieving patients’ debilitating symptoms, vaginal symptoms or candidiasis commonly arise. A holistic approach is essential, and proactive efforts to correct vaginal dysbiosis or optimise the vaginal microbiome may prevent or reduce such side effects.

Future research should build on studies such as those by Sekito et al. [28], utilising modern high-throughput sequencing techniques and diagnostics to further define how agents such as oestrogen or probiotics that have been shown to reduce UTI burden alter vaginal ecology in high-risk groups so that this knowledge can be used to expand treatment options for a condition with a huge impact on quality of life.

## Data Availability

No new data were generated or analysed during this study.
